# An open database on global coal and metal mine production

**DOI:** 10.1038/s41597-023-01965-y

**Published:** 2023-01-24

**Authors:** Simon Jasansky, Mirko Lieber, Stefan Giljum, Victor Maus

**Affiliations:** 1 Vienna University of Economics and Business, Ecological Economics, Vienna, 1020 Austria; 2 International Institute for Applied Systems Analysis (IIASA), Advancing Systems Analysis Program, Laxenburg, 2361 Austria

**Keywords:** Geology, Environmental impact, Databases, Interdisciplinary studies

## Abstract

While the extraction of natural resources has been well documented and analysed at the national level, production trends at the level of individual mines are more difficult to uncover, mainly due to poor availability of mining data with sub-national detail. In this paper, we contribute to filling this gap by presenting an open database on global coal and metal mine production on the level of individual mines. It is based on manually gathered information from more than 1900 freely available reports of mining companies, where every data point is linked to its source document, ensuring full transparency. The database covers 1171 individual mines and reports mine-level production for 80 different materials in the period 2000–2021. Furthermore, also data on mining coordinates, ownership, mineral reserves, mining waste, transportation of mining products, as well as mineral processing capacities (smelters and mineral refineries) and production is included.

## Abstract (summary)

**Table:** 

Measurement(s)	terrestrial mining
Technology Type(s)	Data Collection
Sample Characteristic - Environment	land
Sample Characteristic - Location	global

## Background & Summary

The global mining sector has undergone rapid growth in the past two decades, with global production of mineral fuels, metal ores and industrial minerals amounting to 17.3 billion tonnes in 2020, up 52% from 11.3 billion tonnes in 2000. Some commodities, such as iron ore (151%) or aluminium (166%), substantially outpaced that growth rate in the same period^1^. This trend is projected to continue, with metal ores likely being among the fastest growing material categories^2^, partly due to increased demand induced by the green energy transition and electrification efforts^3–5^.

For accurate analyses of the past and current economic and environmental performance of the global mining sector, as well as for predictions of future trends and impacts, a solid data foundation is required. While robust mineral extraction data is available at the national level^1,6^, for the level of individual mine-sites availability of open data is still poor. Current databases are (a) only available with a paid licence (e.g. the S&P Capital IQ Pro Metals and Mining database^7^) or not comprehensive, comprising records (b) for only one country (e.g. mineral production data for Peru) or (c) one material in one country (e.g. coal data by the EIA for the USA^8^), or (d) are limited in time range (e.g. the Global Coal Mine Tracker database^9^).

A standardised accounting framework for production data of individual mines, including compilation worksheets and reporting questionnaires for national statistical offices (NSOs), has been introduced by the UNEP in 2021^10^, based on the work of West *et al*.^11^. However, such official data compilation will not result in data about past production, and it will take many years, until a large number of NSOs have implemented and published such data collections and records in a comprehensive manner. Considering these limitations of existing mining databases, and the uncertain outlook on future mining data compilation by NSOs, we contribute to filling this knowledge gap by presenting an open database on global coal and metal mine production^12^.

Our open database on global coal and metal mine production^12^ covers worldwide mining activities of metal ores and coal, on an individual mine level. It comprises 1171 mines, production data for 80 different materials, for the time period of 2000–2021. The data was gathered manually from more than 1900 publicly available sources, primarily consisting of publications by mining companies, such as annual or sustainability reports. The database is fully transparent, as all data points are linked to their respective source documents. After manual screening and entry of the data, automatic cleaning, harmonization and data checking was conducted, in order to generate a comprehensive and consistent database. Geo-information of individual mines and processing facilities was obtained either from coordinates available in company reports, or by retrieving the coordinates via Google Maps API^13^, with subsequent manual checking. For mines for which no coordinates could be found, other geographic attributes such as province, region, district and municipality were recorded. Furthermore, all mines were mapped to the Database of Global Administrative Boundaries (GADM)^14^, which delivers highly detailed information on the extent of sub-national regions for countries world-wide, either based on their coordinates, or on the other collected geographic attributes.

Our novel and open-source database enables a wide spectrum of global, multi-commodity, multi-year analyses on mining activities at the spatially explicit level of individual mines. Among others, its applications include the modelling of global raw material flows, environmental assessments of the mining sector, socioeconomic analyses of growth and employment impacts in mining regions, or studies on the origins of critical raw materials. Furthermore, it provides a data foundation for anyone analysing mining activities on the national and sub-national level. In case very high production coverage in a certain region of interest is required for a specific analysis, the database can easily be extended via the framework described below. Thus, our database adds value by both providing a comprehensive, global and open database on mine-level coal and metal production that is directly usable for analyses, and by providing a data foundation that can be easily extended for analyses that require very high production coverage in a specific region.

## Methods

The open database on global coal and metal mine production^12^ is exclusively based on publicly available information. The sources consist primarily of publications of mining companies, such as annual, quarterly or sustainability reports available as online PDF documents, and to a lesser extent of information available directly on the websites of mining companies. We systematically screened these sources, and manually extracted relevant information into a spreadsheet file with a predefined accounting framework (see below for details). The main objective was to gather data on the general properties of mines, and on their physical extraction and production amounts. Additional information, such as data on mineral refining and smelting, transport of mining products, mining waste production, and mineral reserves, was also recorded, if it was accessible in the screened sources. The manually entered raw data was then subject to computational post-processing, yielding a consistent final data product.

### Accounting framework for mine-specific data

As data on global mining encompasses a broad range of information, and a multitude of potential metrics and statistics to collect, the scope of the final data product must be clearly defined. In order to record all information in a detailed and consistent format, we developed an accounting framework for mine-specific data, structured as a relational database, and implemented in nine separate tables. Relevant information on mining was divided into two categories.

The first category comprises information on the general properties of individual mines, including their name, location, mined materials, mining methods, processed materials, and historical information such as opening and closure dates. This data typically neither changes over time, nor does it refer to a specific time period only, making it *time-independent*. To record that time-independent information, a two-level accounting system for mine-sites was implemented: On the top-level, mines are organized in “facilities”, each of which can have several sub-entities called “sub-sites”. A sub-site can be an individual mining pit, an underground shaft, a mineral refining plant adjacent to the mine, or similar, thus a facility represents the combination of all its sub-sites. The rationale for this two-level accounting system is that the structure of reported extraction and production data by mining companies is hardly consistent over time. For some years, data for each sub-site might be available, while for other years, only aggregated data at the facility-level is available. This can be caused by changes in the reporting practices of a mining company, or by a change in ownership of a facility or sub-site. The two-level accounting structure allows to record detailed information for each sub-site for years it is available, while still being consistent on an aggregated facility level.

The second category comprises data that can be attributed to a certain time period. Information on extraction and production, transportation, mineral processing, reserves and resources, and economic data such as ownership and operator structure of a mine, belongs to this *time-dependent* category. Time-dependent information was only collected by year, but not for any shorter time periods.

The most important thematic area of this second, time-dependent category is data on material extraction and mineral production. In the accounting framework, data on the extraction and production of coal, metal ores, metal concentrates, and non-metallic minerals is collected. Entries of metal ores and coal are further classified into either (a) “extracted”, i.e. the mass of usable material extracted in a given year (ROM), or (b) “processed/produced”, i.e. the mass of usable material entering the benefication process, or, if the material does not require benefication (e.g. many types of coal), the mass of usable material that is ready to be shipped. The values of “material extracted” and “material processed/produced” can be different, as material extracted might not be concentrated immediately, but temporarily stockpiled. Furthermore, data on the valuable contents contained in metal ores and concentrates is collected. Here, the relevant variables are: the presence of valuable materials (=commodities) in the ore or concentrate, their mass, associated grade, and their material-specific recovery rate during the concentration process. Additionally, data on mining waste is collected, comprising the production of overburden and waste rock, tailings, as well as total material mined (waste rock + ore mined) and the stripping ratio (mass of waste removed divided by mass of ore extracted^15^).

The relevance of the collected variables and relations among these has been derived from the material flow accounting (MFA) framework proposed by *West et al*.^11^ and recommended in UNEP’s *Manual on Economy Wide Material Flow Accounting*^10^, in order to be consistent with the national-level accounting of mine production. However, to adapt the framework to the mine-specific level, some changes were introduced. The most notable differences are that in our accounting framework for mining data, (a) data is recorded by mine and not by “ore stream” (one mine can have several “ore streams” with different ore characteristics, see *West et al*.^11^ for details), and that (b) values for run-of-mine (ROM), valuable content (commodities), and waste have been separated into three different tables in order to preclude any double entry of data points. The reason for (b) is that even though data on extracted material, contained commodities and generated waste could generally be collected and accounted together in one table, it is usually reported in different levels of detail, e.g. waste production is only available for a whole mine, while ore production comprises different kinds of material. The developed table structure therefore precludes any possibility of double entries, which occurs when recording related variables with different levels of detail in the same table.

However, due to the separation into different tables, it is necessary to relate them. Therefore, entries in the table storing the valuable contents of an ore can refer to an entry of the table storing the extracted material, thereby specifying the commodities contained in a recorded ore or concentrate. Nevertheless, entries of valuable contents can also exist independently, if only data on the commodities contained in an ore/concentrate is reported, but not the amount of ore/concentrate itself. The linking of these two tables is described in detail in the section *Data records* as the relation between the final data tables *minerals* and *commodities*.

Furthermore, the other thematic areas of the second, time-dependent information category are data on minerals processing, transportation of mining products, mineral resources and reserves, and economic metrics. Each of these areas is recorded in a separate table. For mineral processing at refineries and smelters, primarily the type and mass of input and output materials is of interest. If available, also head grade and recovery rate are recorded. For information on transport between mines, processing facilities, harbours, and other logistics hubs, the variables of interest are: type and mass of material, origin, destination, and transport mode. Regarding mineral resources and reserves, only data on reserves, but not on resources, was collected in the accounting framework. The rationale is that relevance was given only to those minerals which can be considered to be exploitable in an economical manner at the current state or in the near future, which is not the case for resources. The amounts of reserves exclusively refer to the sum of proven and probable reserves, as these are subject to a comparatively high likelihood of being mined, whereas possible reserves were not included. Additionally, we collected data on the output capacities of processing facilities. Here, each distinct output capacity value is only recorded for the latest relevant year, implying the same value for all previous years. Finally, on the economic dimension, data on the ownership and on operating companies at mine-sites was collected.

A multitude of other mining-related information exists, which was deemed outside the scope of our database. This includes, but is not limited to, financial data such as costs and revenue, employment data, mine safety metrics such as the fatal injury rate, supply contracts, or geological models of mineral deposits. Furthermore, only data on industrial mines was collected, while data on artisanal or small-scale mining was not.

It must be noted that our accounting framework is primarily designed to record material extraction data of industrial mines employing conventional mining methods, while its practicability is limited for mines relying on leaching. The reason is that leaching processes can take several months to extract metal from an ore, thus making ore grades, recovery rates and metal recovered less meaningful if examining it for only one year instead of over the time span of several years^16^. We recorded instances of leaching where ore is excavated, such as heap leaching, but disregarded *in-situ* leaching, where ore is left in the ground^16^. Due to these limitations, all entries of ores subject to leaching, and the related amounts of extracted metals, were specifically marked in a separate column.

### Online research

In the online research process, conducted from March 2019 to September 2021, data was gathered from more than 1900 source documents. These primarily consisted of annual, quarterly and sustainability reports as well as the websites of mining companies. All data points were linked to their source documents, identified by the publisher, year and title of the publication, e.g. “*Rio Tinto (2019) Annual Report 2018*”, as well as the URL to the report. To access company reports that were not available on the most recent version of a mining company’s website, we screened the history of the respective domain via the online portal archive.org, and retrieved reports from there. A substantial number of company reports were also retrieved from the online portal AnnualReports.com and the Edgar database by the SEC^17^. In cases where reports were not available from those sources, we performed simple Google searches with the company name, report title and year as keywords. Some of these searches resulted in company reports made available by various online portals, such as SINA Finance (finance.sina.com.cn). It must be noted that not all recorded URLs are stable over time. The URLs were recorded at the time of access during data entry, but their availability might change in the future.

### Systematic review of mining company reports

To ensure the greatest initial returns, a ranking of the largest global mining companies, according to their annual ore extraction volume as reported in the S&P Capital IQ Pro Metals and Mining database^7^ for the year 2017, was created. Starting with the company with the greatest ore extraction volume, we visited the company website, downloaded relevant reports, and entered relevant information into the spreadsheet. The first six companies investigated, in order of decreasing amount of ore extracted, were Newmont Mining Corporation (USA), Southern Copper Corporation (USA), Barrick Gold Corporation (CAN), Kinross Gold Corporation (CAN), AngloGold Ashanti Limited (ZAF), and Rio Tinto (GBR). Additional to this ranking, various public websites listing large mining companies, e.g. MINING.COM, Wikipedia, or Mindat.com, were evaluated.

Mines and mining concessions are frequently traded between mining companies. When this was encountered during online research, the companies on both sides of the transaction were screened, and relevant information was integrated. This ensures capturing the longest possible production timelines for individual mines, regardless of ownership changes. Reports that contained relevant information, but were published in other languages than English, were translated with Google Translate. This was particularly utilized for reports of Chinese coal mining companies.

### Limitations of online research

The greatest obstacle of online research relying on publicly available company reports is missing information, which in most cases simply corresponds to information not made public by mining companies. A significant gap in available information was identified between publicly listed mining companies, publishing more detailed information, and private or state-owned companies, publishing little or no information.

While missing information presents the largest obstacle to construct a consistent mining database with high global coverage, also information that is published in company reports was often found to be incomplete or ambiguous. Regarding ambiguous reporting, we identified three dimensions: Arbitrary aggregation of elements, arbitrary aggregation of data for several mines, and ambiguous unit specifications.

First, the dimension of arbitrary aggregation of elements typically arises when production values of several metals contained in a poly-metallic ore are aggregated, and only one number is published for all metals. This can take the form of reporting more than one metal together as “equivalents”. For example, in a poly-metallic ore containing gold and silver, the silver is converted to “gold equivalents” based on the spot prices for both metals^18^, and only a combined production value for “gold equivalents” is stated. Another form is the reporting of several elements contained in a poly-metallic ore as a “bundle” of elements. For example, for a poly-metallic ore containing the elements platinum, palladium, rhodium, ruthenium, iridium, and osmium, the company report only specifies the aggregated grade and production of all these elements as “platinum group metals (PGM)”^19–21^. Both these practices make it impossible to derive the ore grade and the mass of each individual metal. To further highlight the heterogeneity of mining data reported in company reports, different companies also have different definitions of platinum group metals: While the elements platinum, palladium, rhodium, ruthenium are part of the definition for “6E PGM” of all three of the following companies, *Sibanye Gold* also includes osmium and gold^22^, *African Rainbow Minerals* includes iridium and gold^19^, and *Norilsk Nickel* includes osmium and iridium^20^. While reporting in element bundles is not ideal, it was still recorded in the raw data by creating aggregated material categories for “element bundles”, and by explicitly specifying production of “equivalents” in the “comment” column.

Second, aggregation of production was also encountered at the spatial level, where only one number for the production of several mines was reported. Examples include “The Boliden Area” in Sweden^23^, or the “Yanzhou Shandong Area” in China^24^. The two-level accounting structure of facilities, introduced in section *Accounting framework for mining data*, allows to record such aggregated data in a consistent manner. However, spatial detail of the exact location of extraction is lost.

Third, the reporting of different mass units was often ambiguous, in particular the distinction between short tons, long tons, and metric tons, as well as between standard ( = “avoirdupois”) ounces and troy ounces. Ambiguities arose when only “tons” or “ounces” was stated, which could refer to either of the units. We applied computational data checks to correct false unit entries, see section *Technical Validation* for details.

### Data processing

After the construction of the accounting framework, and its population with data from mining company reports, the obtained spreadsheet contained manually entered, raw data. This spreadsheet served as the input file for the data processing pipeline, at the end of which a consistent, final data product, the open database on global coal and metal mine production^12^, is yielded. The data processing pipeline consists of harmonization, conversion, aggregation, gap-filling, geographic referencing, and restructuring. Additionally, data checks were applied during post-processing, see the section *Technical Validation* for more information. All processing steps were conducted via R scripts^25^, and can be reproduced with the code available at www.github.com/fineprint-global/compilation_mining_data.

#### Step 1: Harmonization

As data was manually entered and thus prone to human errors, columns were checked for non-emptiness and correct data types. Furthermore, information was entered as stated in the company source, leading to several different wordings referring to the same item, such as “Thermal coal” and “Steam coal”, “DRC” and “Democratic Republic of Congo”, or “tonne” and “metric ton”. These heterogeneous entries were harmonized by assigning predefined categories to entries of materials, units and countries via a concordance table.

#### Step 2: Conversion

Various units were converted to standard units via a concordance table. Standard units used are tonnes (t) for mass, parts per million (ppm) for ore grade, and square kilometres (sq. km) for area. Furthermore, mines and processing facilities can be jointly owned by several companies. This can result in production being stated as a share of total mine production, attributable to the respective reporting company. The attributable share was recorded in the original data, and total production was calculated by dividing attributable production by the company’s attributable share of total production. During data collection, it was taken into account to only record production values from one company for every jointly owned mine, ensuring not to introduce any double counting.

#### Step 3: Aggregation

To establish consistency throughout production time-series, values were aggregated for each facility, year, and material, thus disregarding distinct values for individual sub-sites and mining methods. To get the aggregated mass of production, a simple sum was calculated, while for the variables grade, yield and recovery rate, a weighted average was calculated, according to the mass of the material they referred to. The rationale behind this aggregation was to reduce complexity in the final database, compared to the rather complex mineral accounting framework, to make the data more consistent and to avoid the introduction of erroneous data during the next step, i.e. the gap-filling. If required for specific analyses, disaggregated, unprocessed data is available in the input spreadsheet “detailed_data_mining.xlsx” on GitHub.

#### Step 4: Gap-filling

If data was available for only one of either “extracted material” or “processed/produced material”, the available value was applied as a proxy for the missing one. If a value for the mass of material sold was available, missing production values were estimated by using the amount of material sold as a proxy value. With regard to metal ores, missing values for ore and metal production, head ore grade, and recovery rate, were estimated based on the relationship of the four variables ore processed (*OP*), metal produced (*MP*), head grade (*HG*), recovery rate (*RR*), as stated in Eq. 1:

1 $$OP=MP\ast HG\ast RR$$

To estimate one of the variables for one mine in one year, the availability of the other three was checked. If they were available, we estimated the fourth by plugging them into Eq. 1. Furthermore, *OP*, *MP*, and *HG* were also estimated, if *RR* was not available, but the other two values were. In such a case, we simply excluded *RR* from Eq. 1, and estimated missing values by applying Eq. 2:

2 $$OP=MP\ast HG$$

This approach was judged to be less error-prone, compared to estimating *RR* with the mean value of all available recovery rates for a specific commodity, considering the complexity and diversity of recovery rates, resulting from the composition of ores with regard to their very specific material contents, and to some extent from the applied processing steps. For proper estimations of *RR*, a sufficiently comprehensive set of sample data of recovery rates would have been required, in order to match comparable ore types and variations of metal contents. However, such data with sufficient detail was not available to the authors at the time. This does not mean that *RR* was assumed to amount to 100 percent, but given the just mentioned reasons, excluding it was considered a less error-prone approach than estimating values based on potentially erroneous *RR* data. Furthermore, several specifications were taken into account during the estimation, for example applying yield instead of HG*RR, or to exclude metal production from leaching. In a final gap-filling step, we estimated missing values of produced metal by using values of payable metal as a proxy. Wherever a data point was estimated, its respective source entry was adjusted to specify the estimated variable.

#### Step 5: Geographical referencing

Wherever possible, facilities were linked to their latitude and longitude coordinates. If coordinates of mines and processing facilities were available directly in a company publication, they were recorded from that source. However, the specification of coordinates was rather the exception. Therefore, also other geographical attributes of facilities stated in company reports, such as country, state, province, region, district, county and municipality were recorded.

Facilities for which no coordinates were specified in the company source were georeferenced by querying the name of the mine, and the other available geographical attributes, to the Google Maps API^13^, in order to retrieve exact coordinates. If coordinates were returned, they were subsequently checked by visual inspection of satellite images on Google Maps. In case no coordinates were returned for a facility, or the coordinates were rejected after visual inspection, we determined an area, such as a state or a district, in which the facility is located, by evaluating the available geographical attributes. In order to standardise these political boundaries, they were mapped to the Database of Global Administrative Boundaries (GADM) version 3.6^14^. This mapping was done via a manually constructed concordance table. To ensure consistency, facilities with coordinates were also mapped to GADM regions by a simple intersection of their coordinates with polygons of GADM regions. For this intersection, polygons of the most detailed GADM layer available for each country were used. However, layer level 5 of the GADM database, the layer with the highest granularity, was disregarded, as it was only available for France and Rwanda^14^.

According to the implemented two-level accounting system, facilities and sub-sites can have coordinates. If a facility does not have any sub-sites, it can only have one single coordinate pair resulting in a point geometry. If a facility has more than one sub-site with coordinates, the coordinates of all sub-sites were united to a multipoint geometry. This multipoint geometry was then assigned as the geometry for the parent facility. Consequently, one facility can have several coordinates, and thus also be linked to several GADM regions. If a facility or a sub-site has no coordinates, an empty point geometry was assigned. Spatial operations were conducted with the R package sf^26^.

#### Step 6: Restructuring

In order to achieve a consistent and clean data format in the final database, restructuring was applied. The first two tables, containing information on facilities and sub-sites, were combined, and a unique ID “facility id” was assigned. Each ID is composed of 11 characters, where the first eight indicate the parent facility, the last two the sub-site, with a point as separator. The first eight characters, uniquely identifying the parent facility, are composed of the string “COM”, indicating that the data is sourced from published company information, followed by a five-digit running integer. The last two digits indicate whether the ID represents a facility or a sub-site: the ID of a parent facility, i.e. the entity that represents all its (children) sub-sites together, always ends on two zeros, while the IDs representing single sub-sites end on a running integer, starting from “01”. For example, in the case of the mine “Ahafo”, a gold mine in Ghana, this results in the ID “COM00009.00” for the facility “Ahafo”, “COM00009.01” for its first sub-site “Ahafo North”, and “COM00009.02” for its second sub-site “Ahafo South”. It must be noted that if a facility has sub-sites, the facility itself (e.g. “Ahafo”) represents only the composition of all its sub-sites (e.g. “Ahafo North” + “Ahafo South”), and does not represent a separate location.

Furthermore, in step 6, mines were categorized by primary commodity. While this is simple for mines that only produce a single commodity, like most coal, iron and aluminium mines, it is more complex for mines producing several commodities, and in particular for poly-metallic mines. Therefore, primary commodity categories of poly-metallic mines were allocated according to the economically most significant metal mined. The economically most significant metal was determined by estimating the total value for every metal produced throughout the whole recorded production period. Total value was estimated by multiplying total production of each metal with its average commodity price during the period 2000–2020. The average metal prices were calculated based on harmonized and smoothed price data from the World Bank^27^, Metalary.com^28^, Bloomberg^29^, and USGS^30^.

In a final restructuring step, obsolete columns were deleted, and specific columns renamed or rearranged. The third table of the accounting framework, storing data on mine extraction and production, was separated into two tables, one for coal data, and one for all other mineral data. Furthermore, the seventh table, storing mineral reserves and processing capacities, was also separated into two tables, one for mineral reserves data, and one for processing capacities data.

## Data Records

**Fig. 1:**
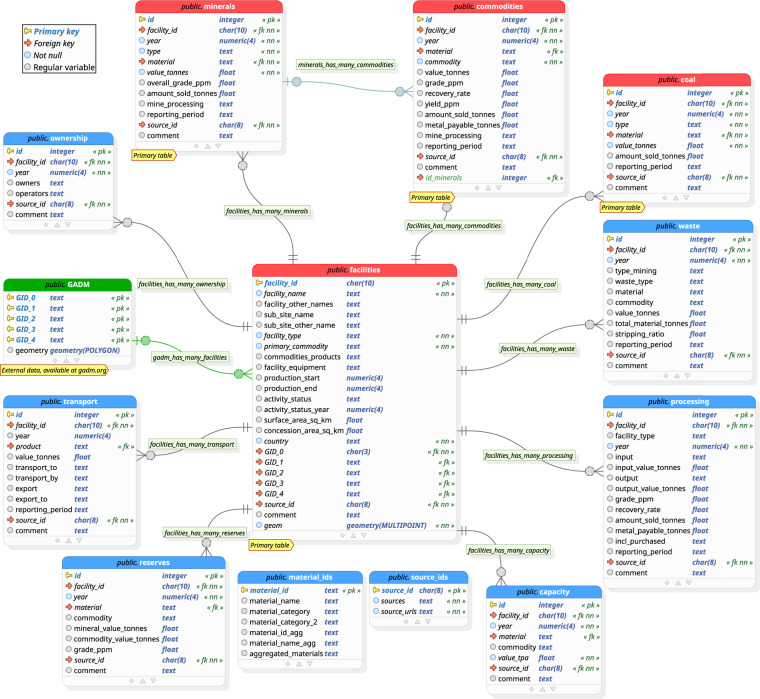
Schematic depiction of the relational database using Crow’s Foot notation. The tables *material_ids* and *source_ids* are referenced by other tables, but graphical connectors are hidden to avoid cluttering the figure. Table *GADM* represents external data, which is available at gadm.org^14^. The first column in every table of the graphic describes the variable name, the second column the data type, the third column indicates optional constraints. Abbreviated constrains are: primary key (pk), foreign key (fk), not null (nn). Graphic created in pgModeler.

The open database on global coal and metal mine production^12^, available on Zenodo under 10.5281/zenodo.7369478, is structured as a relational database with 12 tables. Figure 1 depicts a graphical model of the database, displaying table names, variable names, data types, and relations between tables. The table *facilities* is encoded in GeoPackage format^31^, all other tables are encoded in comma separated values (CSV) format. A description and explanation of all variables can be downloaded as an Excel file from the database repository^12^. All mining coordinates included in the open database on global coal and metal mine production^12^ are displayed on a global map at www.fineprint.global/viewer. Additional information on the general properties and on the production of individual mines is visualised either by clicking on one of the coordinate markers, or at www.fineprint.global/visualisations/global-coal-and-metal-mining-viewer/. In the sections *Data tables* and *Table relations* below, the database structure is described in detail. In section *Geographic coverage*, the spatial representation of the database^12^ and the differences in data availability for different regions are assessed. Section *Production coverage* analyses how much of global and national production of different materials is covered in the underlying database^12^.

### Data tables

The four tables in Fig. 1 with a red header, additionally marked with the label “Primary table”, store core information on the general properties of mining facilities, and data on their physical extraction and production.

#### Table facilities

At the core of the database, and located in the centre of Fig. 1, is the table *facilities*. It holds data on the general properties of mines and processing facilities, and particularly spatial data on their location. If exact coordinates are available, they are stored in the column “geom”. Additionally, all facilities are mapped to the GADM regions they are located in. The keys for this mapping are found in the five “GID” columns. For further details on the mapping of facilities to GADM regions, see the section *Table relations* below. Every row of table *facilities*, storing a unique facility or sub-site, is identified by the primary key “facility_id”. The structure and interpretation of this id was described at the end of the *Data processing* section.

#### Tables coal, minerals and commodities

The other three primary tables *coal*, *minerals*, and *commodities* store all data on physical mine production. As indicated by the names of the tables, table *coal* stores data on the production of various coal types, *minerals* on metal ore and non-metallic mineral production, and *commodities* specifies valuable materials contained in minerals. Every row records an instance of a specific material mined or produced, by a specific facility, in a specific year. Furthermore, every row is uniquely identified by the primary key in column “id”, a running integer starting at one.

#### Other tables

The tables *ownership*, *waste*, *processing*, *capacity*, *reserves*, and *transport* store information that was not the primary focus, but still collected during the data gathering process. Like the tables *coal*, *minerals*, and *commodities*, their names are indicative of the information they store, and every entry (i.e. row) refers to a specific facility and year. The tables *material_ids* and *source_ids*, depicted on the bottom of Fig. 1, do not directly contain information collected during online research, but are supporting tables. Table *material_ids* holds the categorization and nomenclature of various materials. The structure for material ids was developed in concordance with the material categories used for the abiotic accounts for the UNEP IRP Global Material Flow Database^6^. The table *source_ids* stores the sources used during the data gathering process, including publisher, year, title, and URL. As explained in step 4 of section *Data processing*, for estimated data points, the estimated variable is also noted in this table. Finally, as indicated in Fig. 1, table *GADM* represents external data of national and sub-national administrative areas at different granularity levels. This data is not included in the database on Zenodo^12^, but available for download at gadm.org. For the linking of the open database on global coal and metal mine production^12^ with the GADM database^14^, see section *Table relations* below.

### Table relations

Relations between tables are depicted in Fig. 1 by Crow’s Foot notation. Table *facilities* is centrally placed in the graphic, as it is referenced by almost all other tables via the key “facility_id”. All of these relations are of type one-to-many, meaning that one facility can have many entries specifying time-series of extraction, production, mineral reserves, etc.

In order to define the source documents a specific data point is based on, almost all tables reference table *source_ids* via the variable “source_id”. Similarly, the tables *minerals*, *commodities*, *coal*, *waste*, *capacity*, *reserves*, *transport* relate to the table *material_ids*. The foreign keys in these tables are the variables “material”, “commodity”, and “product”, all referencing the primary key “material_id” of table *material_ids*. In Fig. 1, all relations to the tables *source_ids* and *material_ids* are hidden to avoid cluttering the graphic.

As mentioned before, table *commodities* specifies the valuable contents contained in minerals, which is depicted in Fig. 1 by the relation between the tables *commodities* and *minerals*. Here, the foreign key “id_minerals” in table *commodities* references the primary key “id” of table *minerals*. Note that this is a one-to-many relation, as one mineral can contain many valuable materials. Additionally, note that not every entry in table *commodities* must reference an entry in table *minerals*, as it can be the case that data is only available for the mass of valuable content of a mineral, but not for the mass of the extracted or processed mineral itself.

Finally, entries of table *facilities* refer to regions of the GADM database^14^ via the variables “GID_0”, “GID_1”, “GID_2”, “GID_3”, “GID_4”. In case a facility is mapped to more than one GADM region, the “GID” strings specifying the regions are concatenated in the respective “GID” column, with a semicolon as delimiter.

### Geographic coverage

The table *facilities* has 2413 entries, of which 1435 are facilities and 978 are sub-sites. Of the 1435 facilities, 1323 have one or more coordinate pairs specifying their exact location, while 112 do not have coordinates, but are only mapped to GADM regions for spatial determination. Of the 1323 facilities that have coordinates, 1187 have one coordinate pair resulting in a point geometry, and 136 have more than one coordinate pair resulting in a multipoint geometry. In total, 1735 coordinate points specifying the location of mining or mineral processing activities are included in the database.

Furthermore, all 2413 facilities and sub-sites are composed of 2066 mines, 96 smelters, 67 mineral refineries, and 184 entities of other forms. Other forms include combinations of “mine”, “smelter” or “refinery”, but also include the types “region” and “company”. The types “region” and “company” were only recorded if company sources only specified mining output per company or per region, and no other, more detailed information was available. Entries of type “region” and “company” typically do not have exact coordinates, but are mapped to GADM regions.

Facilities are located in 80 different countries. Countries with the most recorded facilities are the USA (233), Australia (213), Russia (100), South Africa (100), China (95) and Brazil (89). The database includes extraction and production values for 80 different materials, of which 6 refer to coal types, 21 to metal ores, 12 to metal concentrates, and 19 to metals. All materials included in the database are stated in the file materials_covered.xlsx on Zenodo^12^.

**Fig. 2:**
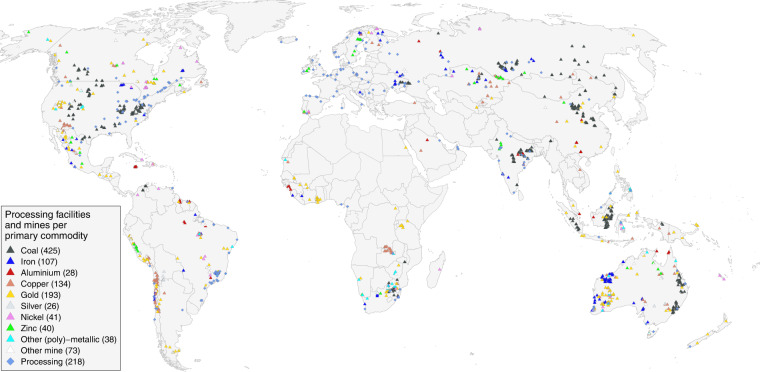
Global map of facilities covered in the open database on global coal and metal mine production^12^. Mines are categorized by their primary commodity. A Robinson map projection is used.

Figure 2 depicts mines and processing facilities with coordinates on a global map. Mines are categorized by their primary commodity production, determined by the column “primary_commodity” of table *facilities*. Figure 2 clearly depicts the spatial agglomeration of mining coordinates in certain areas, such as along the Andes mountain range, in South Africa, Kalimantan in Indonesia, or in Western Australia. However, it is also highlights that some important mining areas are underrepresented. In particular in China, being the largest producer for many mining commodities^1^, and having the second largest mining area globally^32,33^, our open database lacks mining coordinates. This is also reflected in the comparatively low number of recorded facilities in China (95), and the low share of Chinese mining activities covered in the underlying database^12^, as discussed below.

### Production coverage

To assess the extent of total mining production covered in the open database on global coal and metal mine production^12^, its reported production was compared to national production figures. For comparison data, an intermediate data set from the compilation of the UNEP IRP Global Material Flows Database was used^34^. It contains national production data for all abiotic materials, including coal, metal ores and non-metallic minerals. This data set was used because it is the only harmonized data with global coverage and sufficient detail on all materials required for such a comparison (unlike the more aggregated data sets in the official UNEP IRP Global Material Flow Database^6^). Furthermore, the material classification and nomenclature were already similar to the one used in the open database on global coal and metal mine production^12^. However, slightly altering definitions of materials with otherwise the same name and ID were identified to be problematic in the comparison of production values between the two data sets, which is discussed in the Supplementary Material. Furthermore, to calculate shares of total national production covered, it was assumed that production values stated in the comparison data set^34^ represent 100% of the national production. This does not necessarily have to be the case, especially for countries with large unofficial artisanal mining activities.

**Fig. 3:**
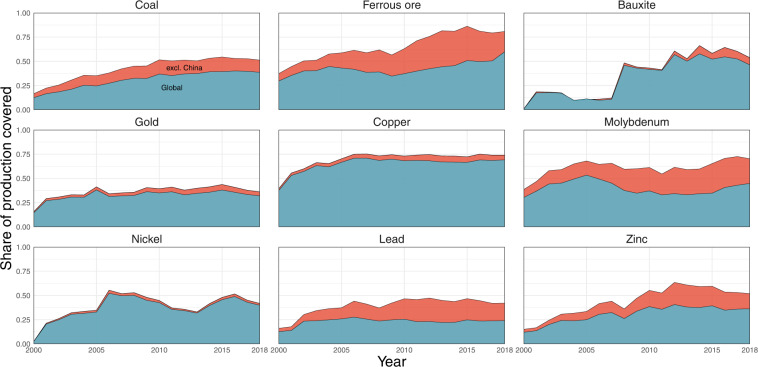
Share of global production covered in the open database on global coal and metal mine production^12^, for the years 2000–2018. The blue layer depicts global coverage, the red layer depicts global coverage excluding China. An intermediate data set from the compilation of the UNEP IRP Global Material Flows Database^34^ was used for the national production comparison values.

Figure 3 depicts the share of global production covered (“coverage”) for nine selected materials in the period 2000–2018. This share was calculated by comparing production data of the open database on global coal and metal mine production^12^ to national production data of the above-mentioned intermediate data set^34^, with data from both being aggregated globally, for every year and material. The period 2000–2018 was chosen because for these years, data availability in both data sets^12,34^ was sufficiently high to produce meaningful coverage shares. The blue layer in Fig. 3 depicts coverage on a global level, while the red layer shows global coverage excluding all extraction values from China, both from the underlying database^12^, and from the comparison data set^34^. The reason for this distinction in the display of coverage is that while China is the largest producer for many mining commodities^1^, the share of Chinese national extraction covered in the open database on global coal and metal mine production^12^ for most materials is significantly smaller than for other countries (see files coverage_table.pdf and coverage_national_area_charts.pdf on Zenodo^12^). This disproportionally low national coverage share is also reflected in Fig. 3, as for almost all materials and years, the global coverage share is higher excluding than including China. The difference in coverage share reaches up to 38 percentage points, which is the case for ferrous ore coverage in the year 2013, when global coverage is 44%, and coverage excluding China is 82%. For some analyses based on the underlying database^12^, it therefore might be reasonable to exclude China as well.

For the first three materials depicted in Fig. 3, coal, ferrous ore, and bauxite, values of ROM production of both data sets^6,12^ were compared. All sub-categories of coal, such as lignite or anthracite, were aggregated in both data sets. Additionally, for individual mines and years where no production of ferrous ore is reported in the open database on global coal and metal mine production^12^, iron concentrate production of the same mine and year is included in the aggregated iron ore production values. This has the benefit to also incorporate cases in the coverage calculations, where a company only reports iron concentrate production, but no ferrous ore ROM production. Iron concentrate production of a mine is always smaller than its ferrous ore ROM production, therefore this procedure might lead to under-estimation of the coverage share, but certainly prevents over-estimation. Other particularities of the iron ore coverage calculations, possibly leading to over-estimation, are discussed in the Supplementary Material.

For the remaining six materials copper, gold, molybdenum, nickel, lead and zinc, values for metal production of both data sets^12,34^ were compared. A comparison of ores is not reasonable, as the underlying database^12^ includes mono- and poly-metallic ores for these metals, while the national production^34^ includes approximations of amounts of ore associated with each of these individual metals based on concentrations and price-based ore allocations (see section 6.4 of the Technical Annex for the Global Material Flows Database^35^ for details).

Significant differences in the global coverage shares exist between materials. In most years displayed in Fig. 3, global copper production coverage consistently lies between 60 and 70 percent, whereas for gold the coverage fluctuates between 30 and 40 percent. Furthermore, the coverage share of one material can significantly vary for different years. For example, bauxite coverage jumps from 11% in the year 2007 to 46% in the year 2008. In this specific case, the reason for the jump in coverage is Rio Tinto’s acquisition of Alcan, a major former bauxite mining company^36^. After this acquisition, bauxite production of former Alcan mines is included in the underlying database, explaining the large increase in coverage. In general, a trend of higher coverage in more recent years is observed for most materials, which can be explained by better accessibility of company reports for more recent years.

**Fig. 4:**
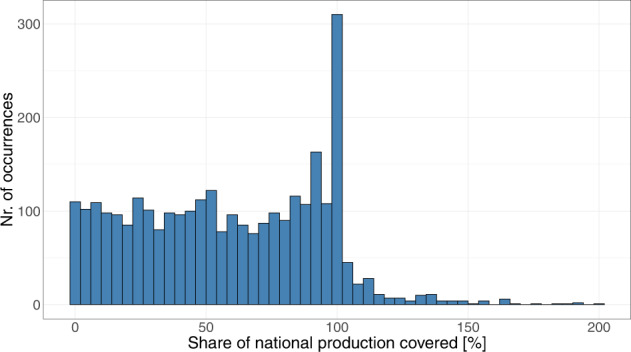
Histogram depicting the distribution of national production coverage shares. Each occurrence represents a coverage share for one country, year and material. Histogram excludes outlier cases with a coverage share larger than 200% (n = 5).

Figure 4 shows the distribution of all coverage shares, each occurrence representing a coverage share for one country, year and material.

Noticeable is a high number of coverage shares around 100%. This suggests that for a large number of countries, materials and years, total national production is fully covered in the open database on global coal and metal mine production^12^. In total, 310 coverage shares are between 98% and 102% (10 percent of all coverage shares), whereas 64 are exactly 100% (2 percent of all coverage shares). Some particularly well covered combinations of countries and materials, where coverage is between 98% and 102% for several years are: Copper, lead and zinc production in Sweden (years 2008–2017), copper production in Argentina (years 2005–2018, except 2014), iron ore production in Brazil (2000, 2015, 2016), or nickel production in Canada (2007–2009, 2012, 2014, 2017, 2018). Furthermore, interesting combinations with a coverage of at least 90% for multiple consecutive years are: Iron ore production in Australia (2001–2018), and copper production in Chile (2003–2018). Thus, the underlying database^12^ is especially well suited for analyses investigating these country-material-year combinations. The occurrence of coverage shares above 100% will be discussed in the next section *Technical Validation*.

Coverage shares for every material and country with recorded production in the underlying database^12^ can be found in the file coverage_table.pdf on Zenodo^12^. Furthermore, figures for the coverage shares of major raw material producing countries, similar in layout to Fig. 3, are available in the file coverage_national_area_charts.pdf on Zenodo^12^.

## Technical Validation

In the process of manually collecting large amounts of data from heterogeneous source documents, a variety of potential sources of error arise. Firstly, information stated in company reports can be false or ambiguous. Surprisingly many instances of ambiguous reporting were encountered, as explained in section *Limitations of online research*. Secondly, human-induced errors during data entry are likely to occur, due to the heterogeneous formats in which mining data is reported, and due to the high number of entered data points. To detect and correct erroneous data, computational data checks were performed during post-processing. Furthermore, to identify potential over-reporting, global and national coverage shares were calculated as explained in the previous section *Production coverage*.

### Data checks

First, simple checks on internal data integrity were performed on the raw input data, consisting of checks for the correct data structure, data type, and potential duplicate entries. Second, a data check based on the consistency between the variables ore processed (*OP*), metal produced (*MP*), head grade (*HG*), recovery rate (*RR*) was performed. The relationship of the four variables was introduced in Eq. 1. This data check aimed to detect both typing mistakes and systematic errors in the unit specification. For every mine and year where all four variables were available, they were plugged into Eq. 1. Then, the value for *OP* (left side of Eq. 1) was compared to the product MP*HG*RR (right side of Eq. 1). In an ideal case, the values on both sides of the equation match exactly, fulfilling the relationship given in Eq. 1. A difference of up to five percent was tolerated, all cases exceeding this tolerance interval in either direction were manually checked. In an initial application, a total of 468 data points were judged to be erroneous, and were subsequently corrected. In the majority of the corrected cases, the unit of metal produced (*MP*) was, due to ambiguous unit specification in company reports, erroneously entered as “avoirdupois ounces”, and corrected to “troy ounces”. Note that this data check was deployed before any gap-filling, described in step 4 of section *Data processing*, was conducted.

### Comparison with national data

Finally, as described in section *Production coverage*, production values of the open database on global coal and metal mine production^12^ were compared to global and national production values. Cases where the coverage share for an individual country, material and year was higher than 120% were manually checked and potentially corrected. In some cases, excessively high coverage was caused by typing errors or the false entry of a unit. However, not all of the cases exceeding 120% coverage were judged to be caused by erroneous data in the open database on global coal and metal mine production^12^, but rather by under-reported production values in the comparison data set^34^. For the case of ferrous ore, where coverage is above 120% for several countries in several years, it was identified to be caused by a slightly different definition of “ferrous ore production” in the comparison data set^34^, which is explained in the Supplementary Material. In other cases of coverage higher than 120%, where after manual inspection no apparent error in the open database on global coal and metal mine production^12^ could be determined, no corrections were made. All cases where national production coverage is above 100% can be obtained from the file coverage_table.pdf on Zenodo^12^. National coverage shares are mainly distributed between 0% and 100% (n = 2738), rarely exceed 100% (n = 279), and even more rarely exceed 105% (n = 143). In percentages, coverage shares are above 100% in about ten percent, and exceed 105% in only about five percent of all cases where coverage can be calculated. This is also reflected Fig. 4, already introduced in section *Production coverage*, displaying the distribution of all coverage shares in a histogram.

## Usage Notes

The open database on global coal and metal mine production^12^, available at 10.5281/zenodo.7369478, is distributed under the licence Creative Commons Attribution 4.0 International (CC BY 4.0). The database is structured as a relational database with 12 tables, where table *facilities*, storing spatial information, is encoded in GeoPackage format^31^, and all other tables are encoded in comma separated values (CSV) format. All other tables can be joined with table *facilities* via the id “facility_id”, linking mine production and other mining data to their geolocation. Such operations can be performed in any software suitable to handle geospatial data, including QGIS^37^, R^25^, and Python^38^.

A description and the datatype of all variables is provided in the Excel file “variables_descriptions.xlsx” on Zenodo^12^. Furthermore, data records are visualised on a global map at www.fineprint.global/viewer, and visualisations of the general properties and production time series of individual mines are displayed at www.fineprint.global/visualisations/global-coal-and-metal-mining-viewer/.

## Supplementary information

Supplementary_Material

## Data Availability

The code used to derive the final data product from the raw input data file is available under the licence GNU General Public License v3.0 (GPL-v3) from the GitHub repository www.github.com/fineprint-global/compilation_mining_data. All processing scripts were written in R^25^, and geoprocessing was conducted with the R package sf^26^.
