# Supplementary material for: An open database on global coal and metal mine production
Source: Sci Data. 2023 Jan 24;10:52. doi: 10.1038/s41597-023-01965-y (PMC9873908; doi:10.1038/s41597-023-01965-y)
Supplement: Supplementary file 1 — Supplementary_Material [file 41597_2023_1965_MOESM1_ESM.pdf]

Supplementary material to the manuscript

"An open database on global coal and metal mine production"

Authors: Simon Jasansky, Mirko Lieber, Stefan Giljum, Victor Maus

1 Considerations for calculating production coverage

Coverage calculations, i.e. determining how much of total global or national mineral production is covered by our underlying database[1], are not trivial and straight-forward. In total, three problems were identified. The first problem is that for most metal ores, available national data accounts[2] are not extensive and detailed enough regarding material types at different processing stages. Therefore, such cases do not allow for a meaningful comparisons on a country-level. This holds especially true for poly-metallic ores and for most metal concentrates. Coverage calculations of these metal ores and concentrates thus cannot be included.

The second problem is that even if comparison values are available for the material and country, there might exist some slight differences in the underlying definitions of the materials, and in the method of compilation for the comparison value. In particular, this applies to ferrous ore data in the comparison data set[2]. USGS, which reports similar values to BGS (the latter being the underlying source for national ferrous ore production in the comparison data[2]), specifies the amount of "usable ore" produced for the USA[3]. In contrast, in the open database on global coal and metal mine production[1], ROM ferrous ore is specified. ROM ore can be subject to beneficiation, reducing its mass and increasing its grade, after which it is specified as "usable ore". Therefore, the values of iron ore production stated in the comparison data set[2], specifying "usable iron ore", will be smaller than the nationally aggregated iron ore production values stated in the open database on global coal and metal mine production[1], the latter specifying ROM ore. This is particularly reflected in the iron ore coverage shares for the USA and Canada, exceeding 100% for several years (see files coverage_table.pdf and coverage_national_coverage_area_charts.xlsx on Zenodo[1]). Furthermore, this difference in the exact specification of ferrous ore production also makes the global coverage share of iron ore less meaningful, as it is potentially over-estimated. To our knowledge, this problem only applies to iron ore production.

The third problem occurs when the time period of reported production, indicated in the open database on global coal and metal mine production[1] in the column "reporting_period", does not match the time period used in comparison data set[2], i.e. the regular calendar year. This problem applies in particular to firms headquartered in countries where the typical financial year deviates from the calendar year, such as Australia (July-June) or India (April-March). In addition, it is not clear, how national and international surveys recording data for deviating periods allocated such data to their standardized accounts based in calendar years. For our coverage calculations, differing reporting periods were disregarded.

References

1. Jasansky, S., Lieber, M., Giljum, S. & Maus, V. Open database on global coal and metal mine production. https://doi.org/10.5281/zenodo.7369478 (2022).
2. Lieber, M. Intermediate data files from the compilation of Economy-wide Material Flow Accounts for the Domestic Extraction of abiotic materials. https://doi.org/10.5281/zenodo.6618340 (2022).
3. USGS. 2018 Minerals Yearbook. Iron ore [Advance Release]. https://pubs.usgs.gov/myb/vol1/2018/myb1-2018-iron-ore.pdf (2021).
